# Metformin, Maternal Glycemic Control, and Neonatal Hypoglycemia After Antenatal Steroids: A Randomized Clinical Trial

**DOI:** 10.1001/jamanetworkopen.2025.52807

**Published:** 2026-01-09

**Authors:** Enav Yefet, Manal Massalha, Gil Talmon, Aminet Labay, Marian Matanis, Erez Sleman, Rima Nassra, Maya Frank Wolf, Inshirah Sgayer, Lior Lowenstein, Zohar Nachum

**Affiliations:** 1 Department of Obstetrics and Gynecology, Tzafon Medical Center, Poriya, Israel; 2 Azrieli Faculty of Medicine, Bar-Ilan University, Safed, Israel; 3 Department of Obstetrics and Gynecology, Emek Medical Center, Afula, Israel; 4 The Ruth and Bruce Rappaport Faculty of Medicine, Technion-Israel Institute of Technology, Haifa, Israel; 5 Neonatology Department, Emek Medical Center, Afula, Israel; 6 Raya Strauss Wing of Obstetrics and Gynecology, Galilee Medical Center, Azrieli Faculty of Medicine, Bar-Ilan University, Safed, Israel

## Abstract

**Importance:**

Although betamethasone reduces complications of prematurity, it can cause maternal hyperglycemia and neonatal hypoglycemia. Metformin effectively treats maternal hyperglycemia and has been shown to decrease neonatal hypoglycemia in women with gestational diabetes.

**Objective:**

To evaluate the impact of metformin treatment after betamethasone administration on maternal glycemic control and the incidence of neonatal hypoglycemia in preterm infants.

**Design, Setting, and Participants:**

This multicenter, open-label randomized clinical trial was conducted from July 1, 2020, to June 30, 2024, at 3 medical centers in Israel. Pregnant women receiving betamethasone from 24.0 to 36.5 gestational weeks due to increased preterm delivery risk were studied. Women with diabetes were excluded.

**Interventions:**

Participants were randomized to metformin (425 mg 3 times daily before meals and 850-1700 mg at 10 pm) or no treatment. The treatment lasted up to 48 hours after the first betamethasone dose. Capillary glucose was measured before meals (preprandial), 90 minutes after starting meals (postprandial), and at 10 pm.

**Main Outcome and Measure:**

The primary end points were the mean maternal glucose values up to 48 hours from first betamethasone injection and the rate of neonatal hypoglycemia in preterm deliveries (<37 gestational weeks).

**Results:**

A total of 169 women (mean [SD] age, 29.7 [5.4] years), including 84 with 48 preterm infants in the metformin group and 85 with 58 preterm neonates in the control group, were included in the study. Mean (SD) maternal total and postprandial glucose values were significantly lower in the metformin group (121 [15] vs 127 [17] mg/dL; *P* = .01; and 129 [22] vs 138 [26] mg/dL; *P* = .009, respectively). Neonatal hypoglycemia rate was lower in the metformin group (10 [21%] vs 23 [40%]; *P* = .04; relative risk, 0.53; 95% CI, 0.28-0.99). Mild adverse effects, mostly gastrointestinal, were reported by 12 women (14%).

**Conclusions and Relevance:**

In this randomized clinical trial, metformin was safe and effective in preventing betamethasone-induced maternal hyperglycemia and neonatal hypoglycemia. Metformin should be considered as a treatment option for women who receive antenatal corticosteroids to prevent their related adverse effects.

**Trial Registration:**

ClinicalTrials.gov Identifier: NCT04332393

## Key Points

**Question:**

Does metformin treatment after betamethasone administration improve maternal glycemic control and reduce the incidence of neonatal hypoglycemia in preterm infants?

**Findings:**

In this randomized clinical trial of 169 pregnant women receiving betamethasone, metformin significantly improved maternal glycemic control and reduced the incidence of neonatal hypoglycemia compared with standard care, indicating a potential therapeutic benefit in this population.

**Meaning:**

Metformin may be a safe and effective strategy to prevent betamethasone-induced maternal hyperglycemia and neonatal hypoglycemia in preterm births.

## Teaser

This randomized clinical trial of pregnant women examines whether metformin can reduce the rate of neonatal hypoglycemia in preterm infants whose mothers receive betamethasone by mitigating maternal hyperglycemia.

## Introduction

Antenatal corticosteroids (ACSs), particularly betamethasone, are routinely administered to pregnant women at risk of preterm delivery due to their well-established benefits, including reduced risks of respiratory distress syndrome, intraventricular hemorrhage, necrotizing enterocolitis, neonatal intensive care unit (NICU) admission, and neonatal death.^1,2,3^ However, ACS administration induces maternal hyperglycemia,^4,5,6^ which in turn contributes to ACS-induced neonatal hypoglycemia.^4,7^

Neonatal hypoglycemia, especially in preterm infants, is a major concern due to its association with serious short- and long-term complications. Short-term complications include neurogenic symptoms (jitteriness or tremors, sweating, irritability, tachypnea, and pallor) and neuroglycopenic symptoms (poor sucking and feeding, weak or high-pitched cry, altered consciousness such as lethargy or coma, seizures, and hypotonia), as well as apnea, bradycardia, cyanosis, and hypothermia.^8^ Additionally, NICU admission may be required, leading to early separation of the newborn from the mother. Neonatal hypoglycemia causes neuroglycopenia at a time when the metabolic demands of the developing brain are high, increasing the risk of brain injury and long-term neurological effects. It has been associated with various brain abnormalities detectable on ultrasonography and magnetic resonance imaging,^9^ and accumulating evidence suggests that even transient and adequately treated neonatal hypoglycemia may be linked to adverse developmental outcomes. Among common neonatal morbidities, it was the only one, including respiratory morbidity, associated with developmental delay at 4 years of age.^10^ A previous systematic review and meta-analysis^11^ reported significant associations with impairments in visual-motor and executive functioning in early childhood and with language and literacy in middle childhood. Given the magnitude of this issue, some experts have suggested that the risks and benefits of ACS administration should be weighed more carefully.^12^

Pregnant women with diabetes are routinely monitored and treated for hyperglycemia after ACS administration. In women without diabetes, insulin effectively reduces ACS-induced hyperglycemia after betamethasone administration; however, the impact on neonatal outcomes has not been assessed.^6^ Although antenatal betamethasone is known to induce both maternal hyperglycemia and neonatal hypoglycemia, current evidence is insufficient to determine whether treating maternal hyperglycemia reduces the risk of neonatal hypoglycemia, particularly in preterm infants.

Metformin is an oral agent commonly used to manage maternal hyperglycemia during pregnancy in cases of gestational diabetes. It has demonstrated comparable effectiveness to insulin, with added benefits, such as lower rates of preeclampsia and NICU admissions and greater ease of administration.^13,14^ In gestational diabetes, metformin has been effective not only in controlling maternal hyperglycemia but also in reducing the incidence of neonatal hypoglycemia compared with insulin, suggesting superior efficacy in preventing this complication.^13,14^

We hypothesize that metformin can reduce the rate of neonatal hypoglycemia in preterm infants whose mothers receive betamethasone by mitigating maternal hyperglycemia. In this study, we evaluated the impact of metformin treatment after betamethasone administration on maternal glycemic control as well as its effect on the incidence of neonatal hypoglycemia in preterm infants.

## Methods

### Study Design

This multicenter, open-label, intention-to-treat randomized clinical trial was conducted between July 1, 2020, and June 30, 2024, at the obstetrics and gynecology departments of 3 university-teaching medical centers across Israel (Tzafon, Emek, and Galilee medical centers). The study was authorized by the local institutional review boards of the participating centers. Participants provided written informed consent. The study followed the Consolidated Standards of Reporting Trials (CONSORT) reporting guideline for randomized clinical trials. The trial protocol can be found in Supplement 1.

### Participants

Pregnant women older than 18 years who received betamethasone due to increased risk for preterm delivery represented the study population. Data on race and ethnicity were not collected. Betamethasone was administered from 24.0 to 36.5 gestational weeks. Enrollment took place right before or within 24 hours after the first dose of betamethasone. We excluded women with pre–gestational diabetes and gestational diabetes because those women receive hypoglycemic medications for glycemic control when corticosteroids are administered according to our departmental protocol. We also excluded women with allergic sensitivity to metformin, chronic heart failure, and chronic kidney failure. Women who did not perform a glucose challenge test or glucose tolerance test were also recruited; however, if they did not complete the test or were diagnosed with gestational diabetes, they were removed from the study.

### Randomization and Masking

Participating women were randomly assigned (1:1) without any preadjustment to study groups, using a computer randomization sequence generation program with a block size of 4. The randomization code was stored in a closed study box, in sealed opaque envelopes, until intervention was assigned by the study physicians.

### Procedures

Pregnant women who were at increased risk for preterm delivery received intramuscular injection of 12 mg of betamethasone according to a physician’s discretion. If delivery did not occur, an additional 12 mg of intramuscular betamethasone was administered 24 hours after the first injection as accepted (women who did not receive the second injection were not removed from the study). Before or within 24 hours of the first betamethasone injection, the women were enrolled in the study and were randomized to either the research or control group. The research group received metformin tablets in the following doses: 425 mg before meals (breakfast, lunch, and dinner) and 850 to 1700 mg at approximately 10 pm. The dosage change was protocol driven after the institutional review boards’ request during the initial study approval: the first 36 women received 850 mg and the remainder received 1700 mg at 10 pm once no serious adverse effects were documented. After confirming the absence of serious adverse effects, the institutional review boards approved increasing the metformin dosage to the maximum allowed by the American College of Obstetricians and Gynecologists.^15^ As betamethasone-induced hyperglycemia lasts approximately 48 hours, it was not feasible to adjust the dose during administration. Therefore, to ensure a measurable effect, we used the maximal dose allowed by the American College of Obstetricians and Gynecologists.^15^ Considering the elimination half-life of metformin, which is approximately 5 hours during multiple dosing,^16^ we administered 425 mg before meals and 1700 mg at 10 pm.

Treatment lasted up to 48 hours after the first dose of betamethasone or until discharge or active labor, defined as 6 cm of cervical dilation^17^ (whichever came first). The control group did not receive treatment with metformin.

In both groups, blood glucose was measured according to the following schedule: before meals (preprandial), 90 minutes after starting meals (postprandial), and at 10 pm. The postprandial measurements were taken 90 minutes after meals because this interval was the time when postprandial glucose peaks in pregnant women with and without diabetes.^18,19^ If an additional course of betamethasone was indicated (at least 1 week after the first betamethasone course and before 34 weeks), the same intervention was administered.

### Outcomes

The primary end points were the mean maternal glucose values up to 48 hours from first betamethasone injection and the rate of neonatal hypoglycemia in preterm deliveries (<37 gestational weeks). We chose to focus on the preterm group because hypoglycemia is more prevalent and severe in this population, largely due to the depletion of hepatic glycogen and fat stores.^20^ Secondary outcomes included mean maternal preprandial glucose values, mean maternal postprandial glucose values, and the rate of abnormal glucose values (preprandial values ≥95 mg/dL, 90-minute postprandial values ≥130 mg/dL, and mean glucose >100 mg/dL [to convert to millimoles per liter, multiply by 0.0555]). The rates of cesarean deliveries and operative deliveries were also recorded.

Demographic, background, and obstetrics characteristics were collected. Adverse effects with possible relation to metformin were documented. In addition, for neonates born prematurely, data regarding neonatal metabolic complications that were routinely assessed were collected, including admission to the NICU, umbilical cord pH, Apgar score at 1 and 5 minutes from birth, hypoglycemia (defined as glucose levels <40 mg/dL and 50 mg/dL during the first day and later if not available in the first day, respectively), hyperbilirubinemia (diagnosis depended on gestational age as accepted), phototherapy, birth weight, and head circumference. Both the pediatricians who treated the neonates and those who collected the data were blinded to the study groups’ allocation. Data regarding fetal malformations and developmental disorders, which were diagnosed near delivery, were also collected.

### Statistical Analysis

Adjustment for multiplicity of the primary end points was made using the Holm method described by Khan et al.^21^ Briefly, the Holm correction requires that the significance level (α) for each hypothesis test is adjusted in a sequential manner: the smallest *P* value is tested at α/2, and if significant, the second *P* value is tested at α. For the maternal end point, to demonstrate a mean (SD) difference of 5 (10) mg/dL, 156 women would be required (2-tailed α = .025, power of 80%). This assumption was chosen due to its clinical relevancy. For the neonatal end point for preterm deliveries, in the study by Gyamfi-Bannerman et al,^4^ neonatal hypoglycemia after antenatal betamethasone and placebo were 24% and 15%, respectively. In the same study, approximately 16% of neonates were born at term and all neonates were older than 34 gestational weeks. We hypothesized that the rate of hypoglycemia would be higher in preterm neonates due to stronger effect of betamethasone administration, but treatment with metformin would reduce the risk to the baseline. To show a reduction in preterm neonatal hypoglycemic from 40% in the control group to 15% in the metformin group, a sample size of 98 neonates would be required (2-tailed α = .05, power of 80%). In a twin pregnancy, each twin is analyzed separately. Recruitment was performed until completion of the sample size for both primary end points. Women were included in the analysis if data on at least one primary end point were available.

Categorical variables were compared using the χ^2^ test or Fisher exact test. Continuous variables were compared via the 2-tailed, unpaired *t* test or Mann-Whitney *U* test. The locally estimated scatterplot smoothing (LOESS) nonparametric regression model was used to demonstrate and compare the mean, preprandial, and postprandial capillary glucose levels from enrollment until 48 hours from the first betamethasone injection. If 2 courses of betamethasone were given, the second course was included. The 95% CIs of the LOESS curves are also presented.^22^

Statistical analyses were performed with SAS software, version 9.4 (SAS Institute). Significance was set at a 2-sided *P* < .05.

## Results

**Figure 1.**
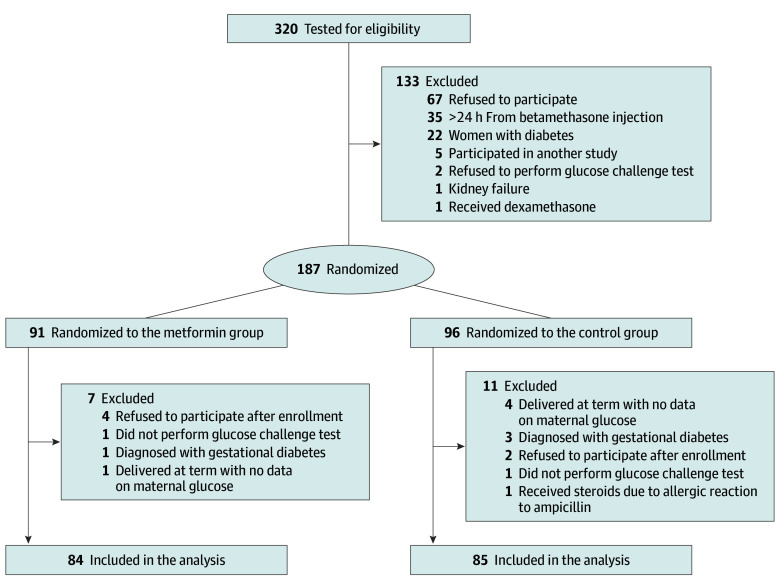
Patient Flowchart

Among 320 women who tested for eligibility, 169 women (mean [SD] age, 29.7 [5.4] years) were included in the study. Of the 169 women, 91 and 96 women were allocated to the metformin and control groups, respectively; and 84 and 85 women in the metformin and control groups were analyzed, respectively (Figure 1). Five women in each group were enrolled in their second betamethasone course. Preterm neonates included 48 neonates from the metformin group and 58 from the control group. Five participants (6%) stopped metformin treatment due to active labor (4 participants) or emergency cesarean delivery (1 participant).

**Figure 2.**
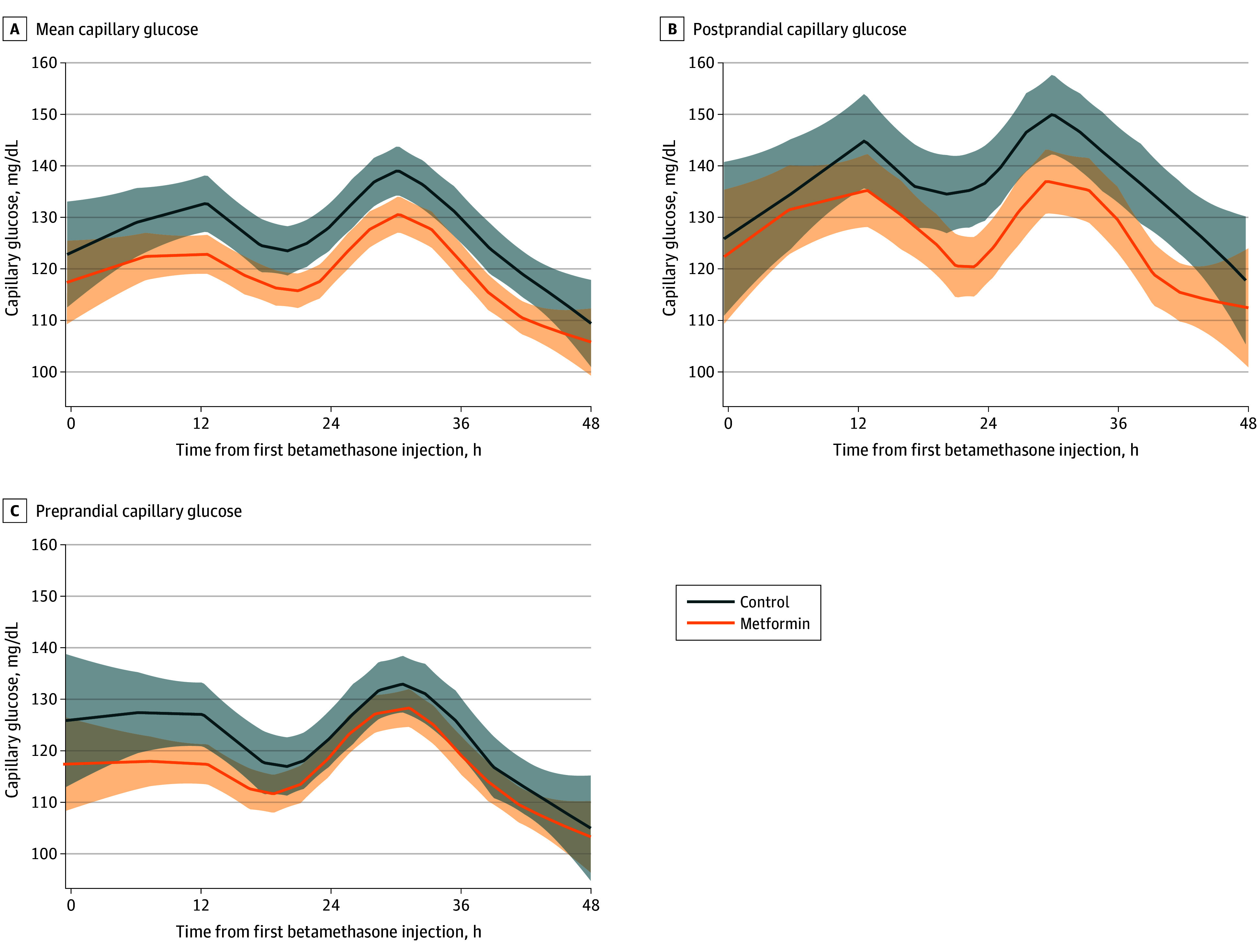
Capillary Glucose Values After the First Betamethasone Injection Locally estimated scatterplot smoothing smooth curve (smoothing parameter, 0.4) with 95% CIs of the mean capillary, preprandial, and postprandial glucose values in the metformin and control groups since the first betamethasone injection. To convert glucose to millimoles per liter, multiply by 0.0555.

Patient and pregnancy characteristics were comparable between the study groups except from higher body mass index (BMI; calculated as weight in kilograms divided by height in meters squared) and higher rate of previous preterm delivery in the metformin group (Table 1). Maternal and preterm neonatal end points are presented in Table 2 and Table 3, respectively. In the metformin group, mean (SD) maternal total glucose values were significantly lower than in the control group (121 [15] vs 127 [17] mg/dL; *P* = .01); and mean (SD) postprandial glucose values were also significantly lower in the metformin group compared with the control group (129 [22] vs 138 [26] mg/dL; *P* = .009) (Table 2) and during the 48 hours after betamethasone first injection (Figure 2). No events of maternal hypoglycemia (defined as glucose ≤60 mg/dL) were documented in either group. The rate of preterm neonatal hypoglycemia was lower in the metformin group compared with the control group (10 [21%] vs 23 [40%]; *P* = .04; relative risk, 0.53; 95% CI, 0.28-0.99). Other maternal and neonatal outcomes were similar between the groups (Table 2 and Table 3). Mild adverse effects were reported by 12 women (14%) who took metformin, mainly gastrointestinal symptoms. Of them, 8 (10%) discontinued treatment due to adverse effects. An additional 5 women (6%) discontinued treatment due to refusal to take metformin without adverse effects. The overall adherence rate was 84%.

**Table 1.** Patient and Study Characteristics^a^

Characteristic	Metformin group (n = 84)	Control group (n = 85)
Age, mean (SD), y	29.8 (5.4)	29.7 (5.5)
Gestational week at enrollment, mean (SD)	31.5 (3.0)	31.9 (2.9)
BMI, mean (SD)	25.9 (5.9)	23.5 (4.9)
BMI ≥30	15 (18)	12 (14)
No. of pregnancies, mean (SD)	2.8 (2.2)	2.6 (1.8)
No. of previous deliveries, mean (SD)	1.1 (1.3)	1.0 (1.2)
Primiparity	36 (43)	38 (45)
Previous preterm delivery	25 (30)	13 (15)
Smoking before pregnancy	5 (6)	7 (8)
Smoking during pregnancy	4 (5)	4 (5)
Marital status		
Married	79 (99)	85 (100)
Single	1 (1)	0
Education duration, mean (SD), y	14.4 (2.8)	14.5 (2.8)
Pregnancy type		
Singleton	77 (92)	76 (89)
Twins	7 (8)	8 (9)
Triplets	0	1 (1)
No. of betamethasone courses from enrollment		
1	72 (86)	75 (88)
2	12 (14)	10 (12)

Abbreviation: BMI, body mass index (calculated as weight in kilograms divided by height in meters squared).

SI conversion factor: To convert glucose to millimoles per liter, multiply by 0.0555.

^a^ Data are presented as number (percentage) unless otherwise indicated.

**Table 2.** Maternal Outcomes

Outcome	Metformin group (n = 84)	Control group (n = 85)	*P* value
Glucose, mean (SD), mg/dL			
Total^a^	121 (15)	127 (17)	.01
Preprandial	115 (13)	119 (16)	.10
Postprandial	129 (22)	138 (26)	.009
No. of glucose measurements, mean (SD)	8.6 (4.5)	8.3 (6.1)	.24
Abnormal glucose measurements, mean (SD), %			
Total	66 (24)	73 (21)	.10
Preprandial	83 (27)	88 (20)	.42
Postprandial	43 (35)	54 (36)	.05
Delivery time, mean (SD), wk	36.0 (3.2)	35.7 (3.1)	.60
Preterm delivery, No. (%)	42 (50)	50 (59)	.25
Delivery type, No. (%)			
Spontaneous vaginal	47 (56)	53 (62)	.12
Vacuum	1 (1)	5 (6)	
Cesarean	36 (43)	27 (32)	
No. of maternal admission days, mean (SD)	6.5 (6.2)	7.3 (8.1)	.88
Labor induction, No. (%)	26 (31)	23 (27)	.58

SI conversion factor: To convert glucose to millimoles per liter, multiply by 0.0555.

^a^ Glucose measurements were available for 83 and 78 women in the metformin and control groups, respectively.

**Table 3.** Outcomes of Neonates Born Preterm^a^

Outcome	Metformin group (n = 48)	Control group (n = 58)	*P* value
Neonatal hypoglycemia^b^	10 (21)	23 (40)	.04
Sex			
Male	26 (54)	32 (55)	.92
Female	22 (46)	26 (45)	
Birth weight, mean (SD), g	1991 (545)	2063 (613)	.53
Time from betamethasone administration to delivery, mean (SD), d	19.0 (17.6)	15.1 (12.4)	.48
Apgar score, mean (SD)			
1 min	8.1 (1.7)	8.3 (1.6)	.54
5 min	9.3 (1.3)	9.5 (0.7)	.98
Apgar score <7 at 5 min	1 (2)	0	.45
Cord pH, mean (SD)	7.3 (0.1)	7.3 (0.1)	.91
Cord pH <7.0	2 (4)	3 (6)	>.99
Overall No. of neonatal admission days, mean (SD)	22.5 (23.1)	18.5 (16.7)	.70
NICU admission	32 (67)	44 (76)	.30
Maximal bilirubin, mean (SD), mg/dL	11.1 (2.5)	11.2 (2.5)	.95
Hyperbilirubinemia	32 (67)	42 (72)	.52
Phototherapy	28 (58)	39 (67)	.34
Head circumference, mean (SD), cm	30.2 (2.4)	30.8 (2.4)	.31
Transient tachypnea of the newborn	3 (6)	1 (2)	.33
Respiratory distress syndrome	6 (13)	7 (12)	.95
Ventilatory support	15 (31)	17 (29)	.83
Supplemental oxygen	14 (29)	10 (17)	.14
Chronic lung disease	3 (6)	2 (3)	.66
Intraventricular hemorrhage	3 (6)	2 (3)	.66
Neonatal sepsis	2 (4)	1 (2)	.59
Hypocalcemia	0	3 (5)	.25
Hypomagnesemia	0	0	
Polycythemia	0	0	
Malformations	1 (2)	2 (3)	.84

Abbreviation: NICU, neonatal intensive care unit.

SI conversion factors: To convert glucose to millimoles per liter, multiply by 0.0555; bilirubin to micromoles per liter, multiply by 17.014.

^a^ Data are presented as number (percentage) of neonates unless otherwise indicated. There were 6 and 7 pairs of twins in the metformin and control groups, respectively.

^b^ Defined as blood glucose less than 40 mg/dL during the first day of life.

## Discussion

This multicenter randomized clinical trial aimed to evaluate the effects of metformin on maternal glycemic control and the incidence of neonatal hypoglycemia in preterm neonates after betamethasone administration. The study found that metformin significantly improved maternal glycemic control and reduced the incidence of neonatal hypoglycemia in preterm infants, suggesting a potential therapeutic benefit for this population.

The study findings indicate that metformin was effective in managing hyperglycemia induced by betamethasone in pregnant women at risk of preterm delivery. Maternal mean and postprandial glucose levels were significantly lower in the metformin group compared with the control group, which aligns with existing literature on the efficacy of metformin in treating gestational diabetes.^13,14^ This finding is clinically important because maternal hyperglycemia has been directly linked to an increased risk of neonatal hypoglycemia, which can lead to severe neonatal complications.^4,7,8,23,24^ By addressing maternal hyperglycemia, this study provides evidence of a preventive approach that could be integrated into clinical practice. Notably, in recent large meta-analyses,^25,26^ exposure to metformin in utero was not associated with adverse metabolic or neurodevelopmental outcomes.

The primary neonatal end point, the rate of hypoglycemia among preterm neonates, was significantly reduced in the metformin group (21%) compared with the control group (40%). This reduction is notable because it supports the hypothesis that improving maternal glycemic control with metformin can mitigate the risk of neonatal hypoglycemia. This finding is particularly important given the association of neonatal hypoglycemia with adverse neurodevelopmental outcomes, as highlighted in previous studies.^8,9^

Metformin was well tolerated, without hypoglycemic events and with only mild gastrointestinal symptoms being the most common adverse effects. In general, we found 14% adverse events rate, which is low and consistent with previous studies.^27,28^ Adherence was high and reached 84% of women. On the basis of the study results, we assume that women will be even more motivated to continue metformin due to its beneficial effects on neonatal hypoglycemia.

The BMI and the rate of previous preterm deliveries were slightly higher in the metformin group. Higher BMI may lead to more severe hyperglycemia due to increased insulin resistance; therefore, the positive effect of metformin may actually be underestimated. The rate of previous preterm deliveries may impact the rate of neonatal hypoglycemia; however, because the delivery week was similar between the groups as well as the rate of preterm deliveries, this potential imbalance is unlikely to influence the effect of metformin. Only 5 participants (6%) stopped metformin treatment due to delivery. As this represents a small portion of the cohort, the modifying effect on the results is minor and likely underestimates metformin effect as well.

The findings from this study suggest that metformin could be considered a standard intervention for managing betamethasone-induced hyperglycemia in pregnant women, with the potential to reduce the incidence of neonatal hypoglycemia. This finding has important implications for clinical practice, particularly in settings where preterm delivery is anticipated and ACS administration is routine.

Future research should focus on the optimal dosing and timing of metformin administration relative to ACSs. Additionally, studies comparing metformin with other glucose-lowering interventions, such as insulin, in this context would be valuable to further delineate the most effective and safe approach for both maternal and neonatal outcomes.

### Strengths and Limitations

A major strength of this study is its randomized clinical design, which enhances the validity of the findings. Additionally, the multicenter approach increases the generalizability of the results across different clinical settings.

However, this study also has limitations to consider. The open-label design may have introduced bias, although the objective nature of the primary end points as well as the fact that both the pediatricians who treated the neonates and those who collected the data were blinded to the study groups’ allocation mitigate this concern. Furthermore, the sample size, although adequate for detecting differences in the primary end points, may limit the ability to detect differences in secondary end points or in subgroups, such as women with varying degrees of hyperglycemia. Additionally, the first 36 women in the metformin group received 850 mg at 10 pm, while the remaining participants received 1700 mg. Subgroup or sensitivity analysis was not feasible due to the small number of participants in each subgroup, and future studies should address the optimal metformin dosage.

## Conclusions

This study found that metformin is safe and effective in preventing ACS-induced maternal hyperglycemia and neonatal hypoglycemia. Metformin should be considered as a treatment option for women who receive antenatal corticosteroid to prevent their related adverse effects.

## Notes

Supplement 1.
Trial Protocol

Supplement 2.
Data Sharing Statement
