# Supplementary material for: Metformin, Maternal Glycemic Control, and Neonatal Hypoglycemia After Antenatal Steroids: A Randomized Clinical Trial
Source: JAMA Netw Open. 2026 Jan 9;9(1):e2552807. doi: 10.1001/jamanetworkopen.2025.52807 (PMC12789950; doi:10.1001/jamanetworkopen.2025.52807)
Supplement: Supplement 1. — Trial Protocol [file jamanetwopen-e2552807-s001.pdf]

Study protocol

Study number: 0021-20-POR

The effect of metformin in pregnant women who received antenatal corticosteroids on glycemic control and the rate of neonatal hypoglycemia – multicenter prospective randomized, controlled trial

Introduction

Antenatal corticosteroids (ACS), particularly, betamethasone is routinely administered to pregnant women at risk for preterm delivery because of a known decrease in risk of respiratory distress syndrome, intraventricular hemorrhage, necrotizing enterocolitis, neonatal intensive care unit (NICU) admissions and neonatal death (1-3). Nevertheless, ACS has been shown to induce maternal hyperglycemia (4-6). In one study, 95% and 77% of non-diabetic women had blood glucose≥140 and ≥160 mg/dL, respectively, at least once during 48 hours following ACS administration. The highest average blood glucose level of 155 mg/dL occurred at 30 hours after administration of betamethasone and the mean maximum blood glucose reached 171 ± 19 mg/dL (5). In another study least squares means of serum glucose was 146 and 162 mg/dL one and two days following ACS betamethasone (6). The indications for antihyperglycemic treatment during pregnancy are pre- and 2 hours post-prandial glucose values of 95 mg/dL and 120 mg/dL, respectively.

The mechanism for ACS-induced maternal hyperglycemia includes antagonizing and decreasing insulin synthesis as well as increasing gluconeogenesis (7). Maternal hyperglycemia contributes to the ACS-induced neonatal hypoglycemia (4;8); the elevation in maternal blood glucose causes higher cord blood glucose and C-peptide levels leading for fetal hyperinsulinemia at birth and consequent hypoglycemia in the early newborn period (9;10). The association between higher maternal serum glucose levels and cord blood C-peptide levels is linear and is demonstrated in all the population including women who are not considered as having gestational diabetes mellitus (10). ACS administration was also shown to cause neonatal hyperbilirubinemia (8).

Neonatal hypoglycemia, particularly in preterm infants, is a major concern which leads to serious short and long term complications. The short term complications include neurogenic symptoms (jitteriness/tremors, sweating, irritability, tachypnea and pallor) and neuroglycopenic symptoms (poor suck or poor feeding, weak or high-pitched cry, change in level of consciousness (lethargy, coma), seizures and hypotonia), apnea, bradycardia, cyanosis, and hypothermia (11). Additional concern is the need for NICU admission causing early separation of baby from his or her mother. Neonatal hypoglycemia results in neuroglycopenia at a time when metabolic activity

is high in the developing brain and so there is a risk of injury and long-term effect. Hypoglycemia has been associated with a variety of brain abnormalities seen on ultrasound and MRI (12) and accumulating evidence now suggests that even *transient and treated neonatal hypoglycemia* may be associated with adverse childhood outcomes. It was the only common neonatal morbidity, including comparison with respiratory morbidity, to be associated with developmental delay at four years of age (13). A recent systematic review and meta-analysis found significant effects on visual-motor function and executive function in early childhood and on language/literacy in mid-childhood (14). The problem of neonatal hypoglycemia following maternal ACS administration is so substantial that it was suggested that the balance of risk and benefit needs to be closely considered (15).

Pregnant women with diabetes are routinely monitored and treated for hyperglycemia after receiving antenatal steroids. In women without diabetes, insulin treatment following antenatal betamethasone was effective in improving ACS-induced hyperglycemia. Neonatal outcomes were not examined (6).

Although antenatal betamethasone was shown to induce both maternal hyperglycemia and neonatal hypoglycemia, to date, there is insufficient data to establish whether treatment for maternal hyperglycemia will decrease the risk for neonatal hypoglycemia, particularly of preterm neonates.

Metformin is an oral medication that is in common use to treat maternal hyperglycemia during pregnancy in gestational diabetes mellitus with comparable results to insulin and the advantage of less rate of preeclampsia, NICU admissions and ease of administration (16;17). Metformin is an effective treatment for maternal hyperglycemia and also was shown to reduce the rate of neonatal hypoglycemia compared to insulin treatment making it more effective than insulin in preventing neonatal hypoglycemia (16;17).

Metformin was also used in clinical trials in non-diabetic obese pregnant women to improve neonatal outcomes. The doses ranged from 1000 to 3000 mg/d (N=1099 women) starting from the early second trimester until birth, without causing maternal hypoglycemia or significant adverse effects other than gastrointestinal symptoms in some of the studies (18). In the study of Nascimento et al (N=375 women), metformin reduced the rate of cesarean delivery in non-diabetic obese pregnant women compared to control. The rate of drug intolerance was reported to be only 4% (19). In

the study of Dodd (N=524), the rate of adverse effect was similar between the metformin and control groups (20).

We hypothesize that metformin will reduce the rate of neonatal hypoglycemia of preterm neonates that their mothers were treated with betamethasone via reduction of maternal hyperglycemia.

In the present study we will examine the effect of treatment with metformin on maternal glycemic control and hypoglycemia in preterm neonates following maternal betamethasone treatment.

Study aims

1. To study the effect of metformin treatment on glycemic control in pregnant women who received betamethasone
2. To study the effect of metformin treatment on neonatal hypoglycemia in preterm neonates born to pregnant women who received betamethasone

Study hypothesis

Metformin will improve glycemic control of pregnant women receiving betamethasone and will reduce the rate of neonatal hypoglycemia of preterm infants

Study design

Multicenter, open-label, randomized, controlled study.

Number of medical centers: 5

Study population

Screening and enrollment:

Pregnant women receiving betamethasone due to increased risk for preterm delivery from Emek medical center, Baruch Padeh medical center,Assuta Ashdod medical center, Ziv medical center and Galil medical center

Inclusion criteria:

- Pregnant women receiving betamethasone from 24 to 36.5 gestational weeks
- Before or within 24 hours following the first dose of betamethasone
- ≥18 years old

Exclusion criteria:

- Women with pre-gestational and gestational diabetes mellitus (GDM)
- Known allergic sensitivity to metformin
- Known chronic heart failure

- Known chronic renal failure
- Refuse to participate
- Refuse to perform glucose challenge test/glucose tolerance test later on

Criteria for study removal:

- Women who did not perform glucose challenge test/glucose tolerance test will be recruited, however, if they will not complete the test or will be diagnosed with GDM they will be removed from the study
- Refuse to participate after enrollment
- No data on both primary outcomes (women will be included if at least a data on one primary outcome will be available, i.e. either maternal glucose measurements or hypoglycemia assessment in preterm infant).

Study groups, interventions, patients' management and outcomes

Intervention

Pregnant patients that are eligible for the study will be divided into the following groups:

1. Research group, which will receive metformin tablets in the following doses: 425 mg before meals (breakfast, lunch and supper) and 1700 mg (2 tablets) around 22:00. Treatment duration will be up to 48 hours following the first dose of betamethasone, or until discharge or until active labor (whichever comes first).
2. Control group will not receive treatment with metformin.

Follow-up protocol

Pregnant women that are at increased risk for preterm delivery will receive intramuscular (IM) injection of 12 mg betamethasone (celestone) according to physician's discretion. If delivery does not occur, additional IM 12 mg betamethasone injection will be administered 24 hours following the first injection as accepted (women that will not receive the second injection will not be removed from the study and metformin will be administrated). Before or within 24 hours from the first betamethasone injection the women will be enrolled to the study and will be allocated randomly to either the research or control groups as described above.

In both groups blood glucose will be obtained according to the following schedule: before meals (pre-prandial), 90-120 minutes after starting meals (post-prandial) and at 10 p.m. The glucose chart will be filled from enrollment and up to 3-4 days following

the first dose of betamethasone, or until discharge or until active labor (whichever comes first).

If additional course of betamethasone is indicated, the same intervention will be administered.

Time of follow-up: up to 3-4 days following the first dose of betamethasone, or until discharge or until delivery (whichever comes first).

Demographic, background and obstetrics characteristics will be collected. Adverse effects with possible relation to metformin will be documented. In addition, for neonates born prematurely, data regarding neonatal metabolic complications that are routinely assessed will be collected from the neonatal medical charts. Those include: umbilical cord pH, APGAR scores, hypoglycemia (defined as glucose levels< 40 mg/dL and 50 mg/dL during the first day and later on, if data from the first day is not available, respectively), hyperbilirubinemia (diagnosis is depended on gestational age as accepted), birth weight and head circumference.

Data regarding fetal malformations and developmental disorders which will be diagnosed near delivery will also be collected.

Study outcomes

Primary outcome:

1. Mean maternal daily glucose values
2. The rate of neonatal hypoglycemia in preterm deliveries (<37 gestational weeks)

Secondary outcomes:

1. Mean maternal daily pre-prandial glucose values.
2. Mean maternal daily post-prandial glucose values.
3. Percent of abnormal values in the daily glucose chart: pre-prandial values ≥ 95 mg/dl, 90 minutes post-prandial values ≥ 130 mg/dl, 120 minutes post-prandial values ≥ 120 mg/dl and mean daily glucose > 95-100 mg/dl.
4. Rate of cesarean sections and operative deliveries.
5. Neonatal outcomes: admission to the neonatal intensive care unit, Apgar score at 1 and 5 minutes from birth, neonatal hyperbilirubinemia and cord blood pH levels when taken.
6. Neonatal anthropometric measurements (birth weight and head circumference), fetal malformations and developmental disorders near birth.

7. Mean and lowest neonatal blood glucose values in preterm neonates.
8. Maternal adverse effects (e.g gastrointestinal discomfort and allergic reaction).

Study Drugs:

- Name: metformin (metformin Teva, glucophage, glucomin)
- Manufacturer: Teva Pharmaceutical Industries Ltd (metformin Teva, glucophage), Dexcel Ltd, Israel (glucomin). If additional metformin tablets from additional manufacturers become available, they will be accepted as well.

- The Side effects may include - Taste disturbance, nausea, vomiting, diarrhea, abdominal pain, hypoglycemia, bloating/abdominal distention, constipation and lactic acidosis (very rare).
- Storage at room temperature
- Maximal acceptable dose during pregnancy: 3,000 mg/day (21)

This medication was used in a previous study to treat maternal hyperglycemia during pregnancy with high efficacy and safety profiles (17). It was also used in non-diabetic pregnant women without severe significant adverse events (18).

Study randomization

Randomization will be done by computerized software according to power calculation inside sealed envelopes (concealment of allocation). In this way the study will be unbiased.

Statistics

Categorical variables will be compared using Chi-square test or Fisher's exact test. Continuous variables will be compared via the Student's t-test or Mann–Whitney-U test.

Sample size:

We set two primary outcomes. Adjustment for multiplicity of the primary outcomes was made using the method described by Holm (22).

For the maternal outcome (mean maternal daily glucose value):

In order to demonstrate a mean difference of 5 mg% with 10 mg% standard deviation in the mean daily glucose charts 156 women will be required (two tailed

alpha=0.025,power of 80%). This assumption was chosen due to its clinical relevancy.

For the neonatal outcome from preterm deliveries (the rate of neonatal hypoglycemia in preterm deliveries):

In the study of Gyamfi-Bannerman et al (4), neonatal hypoglycemia following antenatal betamethasone and placebo were 24% versus 15%, respectively. In the same study around 16% of neonates were born at term and all neonates were older than 34 gestational age. We hypothesized that the rate of hypoglycemia will be higher in preterm neonates due to stronger effect of betamethasone administration but treatment with metformin will reduce the risk to the baseline. In order to show a reduction in preterm neonatal hypoglycemic from 40% in the control group to 15% in the metformin group, a sample size of 98 neonates will be required. In twins' pregnancy, each twin will be analyzed separately.

Recruitment will be done until completion of the sample size for both primary outcomes.

Since one third of the women are expected to deliver prematurely the estimated sample size+10% drop outs is: 323 women.

Number of participants form this center: 200

Confidentiality:

A participant code will be assigned to each study participant. The information collected about each study participant in the study will be encoded and unidentified. All participants, including physicians, statisticians, and research coordinators undertake to maintain the confidentiality of the study. All material collected will be kept in the research room in obstetrics and gynecology department, in specialized folders in a locked cupboard.

Reference List

1. ACOG Committee Opinion No. 402: Antenatal corticosteroid therapy for fetal maturation. *Obstet Gynecol* 111:805-807, 2008

2. Crowther,CA, McKinlay,CJ, Middleton,P, Harding,JE: Repeat doses of prenatal corticosteroids for women at risk of preterm birth for improving neonatal health outcomes. *Cochrane.Database.Syst.Rev.*CD003935, 2015

3. Roberts,D, Brown,J, Medley,N, Dalziel,SR: Antenatal corticosteroids for accelerating fetal lung maturation for women at risk of preterm birth. *Cochrane.Database.Syst.Rev.* 3:CD004454, 2017

4. Gyamfi-Bannerman,C, Thom,EA, Blackwell,SC, Tita,AT, Reddy,UM, Saade,GR, Rouse,DJ, McKenna,DS, Clark,EA, Thorp,JM, Jr., Chien,EK, Peaceman,AM, Gibbs,RS, Swamy,GK, Norton,ME, Casey,BM, Caritis,SN, Tolosa,JE, Sorokin,Y, VanDorsten,JP, Jain,L: Antenatal Betamethasone for Women at Risk for Late Preterm Delivery. *N.Engl.J Med.* 374:1311-1320, 2016

5. Jolley,JA, Rajan,PV, Petersen,R, Fong,A, Wing,DA: Effect of antenatal betamethasone on blood glucose levels in women with and without diabetes. *Diabetes Res.Clin.Pract.* 118:98-104, 2016

6. Star,J, Hogan,J, Sosa,ME, Carpenter,MW: Glucocorticoid-associated maternal hyperglycemia: a randomized trial of insulin prophylaxis. *J Matern.Fetal Med.* 9:273-277, 2000

7. Khani,S, Tayek,JA: Cortisol increases gluconeogenesis in humans: its role in the metabolic syndrome. *Clin.Sci.(Lond)* 101:739-747, 2001

8. Pettit,KE, Tran,SH, Lee,E, Caughey,AB: The association of antenatal corticosteroids with neonatal hypoglycemia and hyperbilirubinemia. *J Matern.Fetal Neonatal Med.* 27:683-686, 2014

9. Sifianou,P, Thanou,V, Karga,H: Metabolic and hormonal effects of antenatal betamethasone after 35 weeks of gestation. *J Pediatr.Pharmacol.Ther.* 20:138-143, 2015

10. Metzger,BE, Lowe,LP, Dyer,AR, Trimble,ER, Chaovarindr,U, Coustan,DR, Hadden,DR, McCance,DR, Hod,M, McIntyre,HD, Oats,JJ, Persson,B, Rogers,MS, Sacks,DA: Hyperglycemia and adverse pregnancy outcomes. *N.Engl.J Med.* 358:1991-2002, 2008

11. Stanley,CA, Rozance,PJ, Thornton,PS, De Leon,DD, Harris,D, Haymond,MW, Hussain,K, Levitsky,LL, Murad,MH, Simmons,RA, Sperling,MA, Weinstein,DA, White,NH, Wolfsdorf,JI: Re-evaluating "transitional neonatal hypoglycemia": mechanism and implications for management. *J Pediatr.* 166:1520-1525, 2015

12. Burns,CM, Rutherford,MA, Boardman,JP, Cowan,FM: Patterns of cerebral injury and neurodevelopmental outcomes after symptomatic neonatal hypoglycemia. *Pediatrics* 122:65-74, 2008

13. Kerstjens,JM, Bocca-Tjeertes,IF, de Winter,AF, Reijneveld,SA, Bos,AF: Neonatal morbidities and developmental delay in moderately preterm-born children. *Pediatrics* 130:e265-e272, 2012

14. Shah,R, Harding,J, Brown,J, McKinlay,C: Neonatal Glycaemia and Neurodevelopmental Outcomes: A Systematic Review and Meta-Analysis. *Neonatology.* 115:116-126, 2019

15. Groom,KM: Antenatal corticosteroids after 34 weeks' gestation: Do we have the evidence? *Semin.Fetal Neonatal Med.* 24:189-196, 2019

16. Guo,L, Ma,J, Tang,J, Hu,D, Zhang,W, Zhao,X: Comparative Efficacy and Safety of Metformin, Glyburide, and Insulin in Treating Gestational Diabetes Mellitus: A Meta-Analysis. *J Diabetes Res.* 2019:9804708, 2019

17. Nachum,Z, Zafran,N, Salim,R, Hissin,N, Hasanein,J, Gam Ze,LY, Suleiman,A, Yefet,E: Glyburide Versus Metformin and Their Combination for the Treatment of Gestational Diabetes Mellitus: A Randomized Controlled Study. *Diabetes Care* 40:332-337, 2017

18. Dodd,JM, Grivell,RM, Deussen,AR, Hague,WM: Metformin for women who are overweight or obese during pregnancy for improving maternal and infant outcomes. *Cochrane.Database.Syst.Rev.* 7:CD010564, 2018

19. Nascimento,IBD, Sales,WB, Dienstmann,G, Souza,MLR, Fleig,R, Silva,JC: Metformin for prevention of cesarean delivery and large-for-gestational-age newborns in non-diabetic obese pregnant women: a randomized clinical trial. *Arch Endocrinol.Metab* 64:290-297, 2020

20. Dodd,JM, Louise,J, Deussen,AR, Grivell,RM, Dekker,G, McPhee,AJ, Hague,W: Effect of metformin in addition to dietary and lifestyle advice for pregnant women who are overweight or obese: the GRoW randomised, double-blind, placebo-controlled trial. *Lancet Diabetes Endocrinol.* 7:15-24, 2019

21. ACOG Practice Bulletin No. 190: Gestational Diabetes Mellitus. *Obstet Gynecol* 131:e49-e-64, 2018

22. Khan,MS, Khan,MS, Ansari,ZN, Siddiqi,TJ, Khan,SU, Riaz,IB, Asad,ZUA, Mandrola,J, Wason,J, Warraich,HJ, Stone,GW, Bhatt,DL, Kapadia,SR, Kalra,A: Prevalence of Multiplicity and Appropriate Adjustments Among Cardiovascular Randomized Clinical Trials Published in Major Medical Journals. *JAMA Netw.Open.* 3:e203082, 2020
