# Supplementary material for: Metformin, Maternal Glycemic Control, and Neonatal Hypoglycemia After Antenatal Steroids: A Randomized Clinical Trial
Source: JAMA Netw Open. 2026 Jan 9;9(1):e2552807. doi: 10.1001/jamanetworkopen.2025.52807 (PMC12789950; doi:10.1001/jamanetworkopen.2025.52807)
Supplement: Supplement 2. — Data Sharing Statement [file jamanetwopen-e2552807-s002.pdf]

Data Sharing Statement

Yefet. Metformin, Maternal Glycemic Control, and Neonatal Hypoglycemia After Antenatal Steroids. *JAMA Netw Open*. Published January 09, 2026. doi:10.1001/jamanetworkopen.2025.52807

Data
Additional Information: Identifying Data Date of trial registration: March 29, 2020 Date of initial participant enrollment: July 5, 2020 URL of the registration site: ClinicalTrials.gov Clinical trial identification number: NCT04332393
Data available: No

Additional Information
Explanation for why data not available: The data from this study is available from the corresponding author upon a reasonable request and following approval of the institutional review board.
